# Anti-Inflammatory Effect of Very High Dose Local Vessel Wall Statin Administration: Poly(L,L-Lactide) Biodegradable Microspheres with Simvastatin for Drug Delivery System (DDS)

**DOI:** 10.3390/ijms22147486

**Published:** 2021-07-13

**Authors:** Piotr Wacinski, Mariusz Gadzinowski, Wojciech Dabrowski, Justyna Szumilo, Jakub Wacinski, Nathalie Oru, Eric Vicaut, Stanislaw Czuczwar, Janusz Kocki, Teresa Basinska, Stanislaw Slomkowski

**Affiliations:** 1 Department of Cardiology, Medical University of Lublin, Jaczewskiego 8, 20-950 Lublin, Poland; jakub.wacinski9@gmail.com; 2 Centre of Molecular and Macromolecular Studies, Polish Academy of Sciences, H. Sienkiewicza 112, 90-363 Lodz, Poland; mariuszg@cbmm.lodz.pl (M.G.); basinska@cbmm.lodz.pl (T.B.); staslomk@cbmm.lodz.pl (S.S.); 3 Department of Anesthesiology, Medical University of Lublin, Jaczewskiego 8, 20-950 Lublin, Poland; w.dabrowski5@gmail.com; 4 Department of Pathology, Medical University of Lublin, Jaczewskiego 8b, 20-090 Lublin, Poland; justyna.szumilo@umlub.pl; 5 Fernand Vidal Hospital, Universite de Médecine Paris 7, 75006 Paris, France; nathalie.oru@univ-paris-diderot.fr (N.O.); eric.vicaut@Irb.aphp.fr (E.V.); 6 Department of Pathophysiology, Medical University of Lublin, Jaczewskiego 8b, 20-090 Lublin, Poland; stanislaw.czuczwar@umlub.pl; 7 Department of Genetics, Medical University of Lublin, Radziwillowska 18, 20-080 Lublin, Poland; janusz.kocki@umlub.pl

**Keywords:** atherosclerosis, inflammation, endothelium, local drug delivery, biodegradable microspheres, poly(L,L-lactide) particles, biomedical application

## Abstract

Atherosclerosis involves an ongoing inflammatory response of the vascular endothelium and vessel wall of the aorta and vein. The pleiotropic effects of statins have been well described in many in vitro and in vivo studies, but these effects are difficult to achieve in clinical practice due to the low bioavailability of statins and their first-pass metabolism in the liver. The aim of this study was to test a vessel wall local drug delivery system (DDS) using PLA microstructures loaded with simvastatin. Wistar rats were fed high cholesterol chow as a model. The rat vessels were chemically injured by repeated injections of perivascular paclitaxel and 5-fluorouracil. The vessels were then cultured and treated by the injection of several concentrations of poly(L,L-lactide) microparticles loaded with the high local HMG-CoA inhibitor simvastatin (0.58 mg/kg) concentration (SVPLA). Histopathological examinations of the harvested vessels and vital organs after 24 h, 7 days and 4 weeks were performed. Microcirculation in mice as an additional test was performed to demonstrate the safety of this approach. A single dose of SVPLA microspheres with an average diameter of 6.4 μm and a drug concentration equal to 8.1% of particles limited the inflammatory reaction of the endothelium and vessel wall and had no influence on microcirculation in vivo or in vitro. A potent pleiotropic (anti-inflammatory) effect of simvastatin after local SVPLA administration was observed. Moreover, significant concentrations of free simvastatin were observed in the vessel wall (compared to the maximum serum level). In addition, it appeared that simvastatin, once locally administered as SVPLA particles, exerted potent pleiotropic effects on chemically injured vessels and presented anti-inflammatory action. Presumably, this effect was due to the high local concentrations of simvastatin. No local or systemic side effects were observed. This approach could be useful for local simvastatin DDSs when high, local drug concentrations are difficult to obtain, or systemic side effects are present.

## 1. Introduction

Atherosclerosis is a complex process involving the deposition of lipids in the vessel wall, wherein it plays a major role not only in cholesterol accumulation but also in the formation of cholesterol. Atherosclerotic plaques occur, but the first changes inside the plaque occur through various factors, such as stress, infection, and blood pressure increases [1]. As a result of the above processes, pathological changes characteristic of atherosclerosis, such as proliferation of smooth muscle cells of the arterial walls, accumulation of inflammatory cells and the formation of lipid deposits, are produced. Vessel wall inflammation and oxidative stress are major dynamic factors that cause the development of atherosclerosis. Inflammation occurring inside atherosclerotic plaques is dangerous and can lead to plaque rupture where their contents enter the lumen, leading to a cascade of complex biochemical processes and the formation of thrombi within the vessel and local tissue ischemia [1,2].

The endothelium and vessel wall play fundamental roles in the regulation of vascular tone, growth, inflammatory response, coagulation, and thrombocyte adhesion [3,4].

The general purpose of drug targeting is delivering drugs to the right place at the right concentration for the right period. Drugs differ substantially in chemical composition, molecular size, hydrophilicity, and protein binding. The essential characteristics that determine efficacy are complex. The bloodstream has natural access for endothelial drug delivery. However, drugs are rapidly eliminated from the blood by renal clearance, hepatic uptake, and the reticuloendothelial system [5]. Particles prepared from poly(L,L-lactide) are very convenient drug carriers, especially those hydrophobic in nature. Poly(L,L-lactide), a biodegradable polymer, undergoes hydrolysis under physiological conditions into lactic acid, which is the natural metabolite in the body. Thus, particles composed of poly(L,L-lactide) and hydrophilic poly(vinyl alcohol) (PVA) at the surface are very suitable coatings for targeted delivery of hydrophobic simvastatin, which has a low bioavailability (average 5%) [6]. The highest oral bioavailable dose for simvastatin is 0.05 mg/kg (max. oral dose 80 mg per 70 kg body mass per day with 5% bioavailability) [6]. In addition, the drug dispersed in the polymer matrix is liberated during a certain period due to gradual hydrolysis of the polymer. This drug class was designed to act in the liver as its main target [7]. The pleiotropic effects of statins on endothelial functions in patients have been well described, but they are especially difficult to achieve in the targeted part of vessels after percutaneous transluminal angioplasty (PTA) because they are rapidly cleared from blood after the first hepatic uptake [6].

In the present study, a novel system based on biodegradable poly(L,L-lactide) microparticles loaded with simvastatin (SVPLA) was developed for local drug delivery into the vascular endothelium/vascular wall. We tested whether it was possible for HMG-CoA reductase inhibitors such as simvastatin to enter the vascular wall in sufficient amounts—in selected parts of vessels (a few centimeters long)—to remain and act for several weeks after a single intravascular injection without general or local side effects. According to our knowledge and literature reports, this is the first attempt to use biodegradable SVPLA microparticles for the above-described application.

## 2. Results

### 2.1. SVPLA Microspheres

The average diameter of SVPLA microspheres loaded with simvastatin, as determined from SEM images, was 6.40 μm ± SD 3.28 with a dispersity index (defined as the ratio of weight average diameter to number average diameter) equal to D_w_/D_n_ = 1.68 (see Table 1). The dependence of individual fractions of SVPLA particles versus particle diameters is presented in Figure S2.

### 2.2. Evaluation of the Release Rate of SV from SVPLA Particles Investigated In Vitro

**Figure 1:**
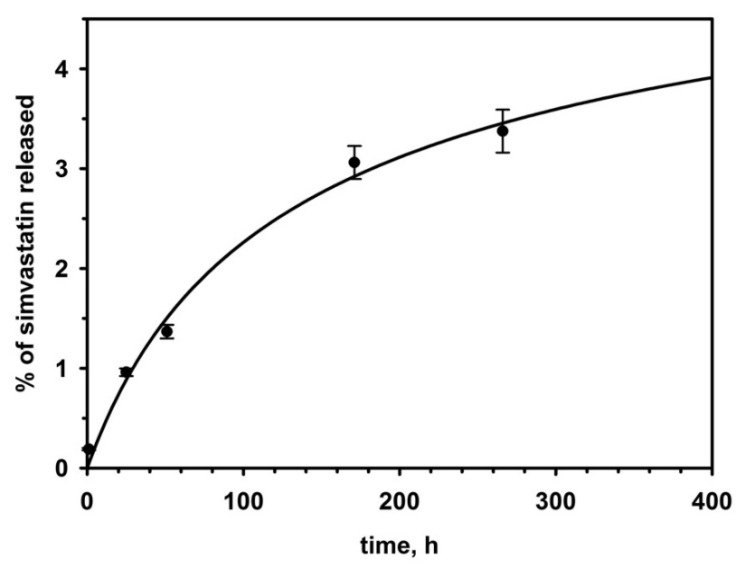
Dependence of the fraction of simvastatin released from SVPLA microspheres versus time.

The dependence of SV released from SVPLA microspheres versus time intervals was tested in vitro. The maximum concentration (plateau) was observed after approximately 350 h. From this assay, the SV release curve of the SVPLA microspheres was calculated (see Figure 1). SV, due to its high hydrophobicity, was exclusively physically bound (by adsorption) with hydrophobic poly(L,L-lactide) chains.

Thus, assuming complete drug release from microspheres, the theoretical simvastatin concentration in the supernatant was equal to 1.4 mg/mL. Taking this value into account, a percentage of simvastatin release was calculated.

Figure 1 presents the dependence of simvastatin release from SVPLA microspheres versus time, investigated in vitro, which is described by the following equation:

y = 6.4335 × (1.0 − 1.0/sqrt (1.0 + 2.0 × 6.4335 × 6.4335 × 0.0001665 × x))(1)

The solubility of crystal simvastatin in water is small, equal to 0.0157 ± 0.004 mg/mL [8]. Thus, considering the drug release from investigated particles, one should expect this value to be a maximal concentration of drug in aqueous medium. However, the obtained slightly higher value (approximately 0.045 mg/mL) may indicate the higher solubility of simvastatin in PBS buffer (pH = 7.4, I = 0.15 M) than in pure water.

### 2.3. Quantitative Assessment of SV Deposition When Administered to a Vessel

The SV concentration in the vessel wall was greatest after the administration of 50% and 25% SVPLA particle dispersions (Table 1 and Table 2).

**Figure 2:**
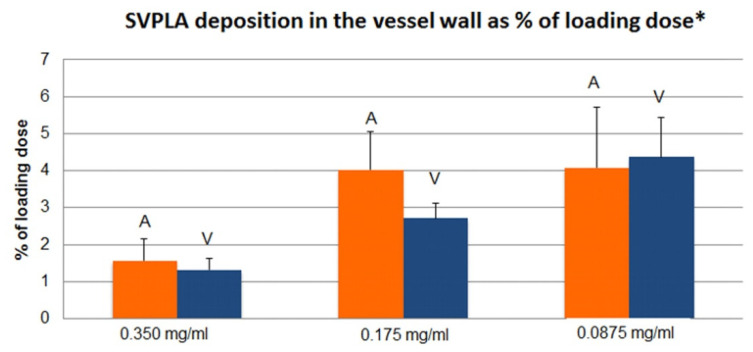
SV deposition in the vessel wall after administration of different concentrations of suspended SVPLA, expressed as a percentage of the total dose administered to the vessel (ia and iv). * *p* < 0.01 vs. control (Control; SV per os; no trace of SV in the vessel wall after p.os administration); A—aorta: V—vein. SV dispersion concentration; A: 0.3500 mg/mL.

SV concentration in the vessel wall was observed when 50% SVPLA was administered into the aorta compared to administration into a vein (*p* < 0.02, as shown in Figure 2). SV deposition in the vessel wall was observed after administration of a suspension of 50% and 25% SVPLA, both intra-arterial and intravenous administration. Deposition accounted for approximately 4% of the total dose of SV administered to the vessel.

### 2.4. Evaluation of Deposition of PLA-DH Microspheres on the Vessel Wall

Fluorescent PLA-DH particles were prepared for visualization of particle adsorption on the surface of vessels. In these studies, we used dansylhydrazine as a fluorescent agent, which was added together with poly(L,L-lactide) during the preparation of particles via emulsification (see Section S2). Based on SEM images, the average diameter and diameter distributions of SVPLA and PLA-DH were similar. Thus, dansylhydrazine did not modify the size and size distribution of SVPLA particles.

**Figure 3:**
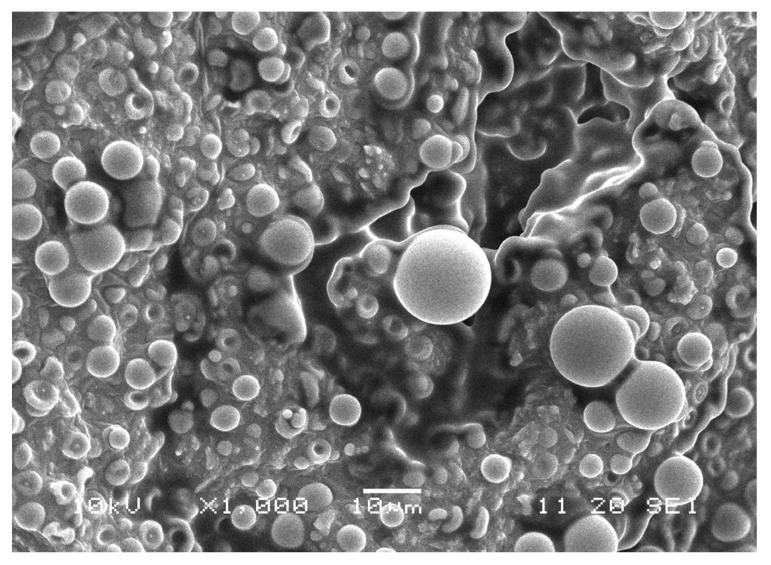
Preparation of rat aorta. PLA-DH microspheres on the surface of the vascular wall. Scanning electron microscopy image (SEM, Jeol 5500LV, Akishima, Japan), 1000× magnification.

**Figure 4:**
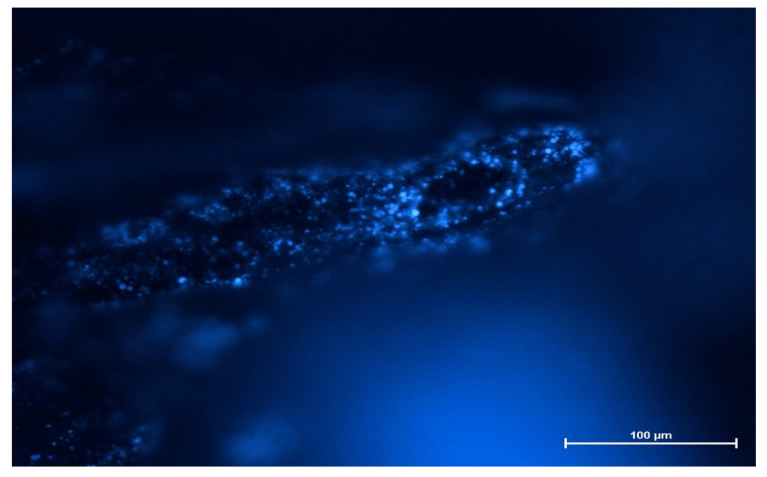
Preparation of rat aorta. PLA-DH microspheres immersed in the aortic wall after administration. Fluorescence microscopy image (Nikon Eclipse TE 2000-S, Japan), 100× magnification.

**Figure 5:**
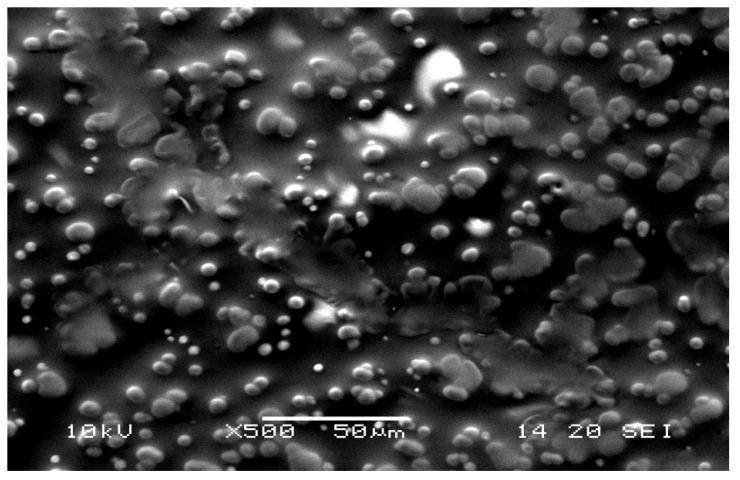
Preparation of rat aorta. Visible microspheres immersed in the vessel wall and a pass. Electron microscopy image (SEM, Jeol 5500LV, Akishima, Japan), 500× magnification.

**Figure 6:**
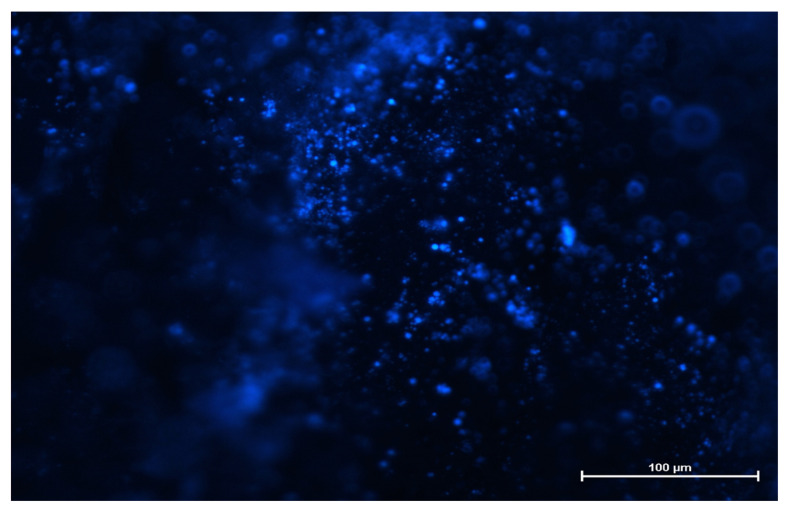
The preparation of rat iliac vein. PLA-DH microspheres visible in the vessel after iv administration. Fluorescence microscopy images (Nikon Eclipse TE 2000-S, Akishima, Japan), 200× magnification.

The fluorescence images revealed that SVPLA microspheres (SVPLA-DH) were displayed in a large quantity on the surface of the vessel, both arterial and venous. The distribution appeared to be relatively uniform. Scanning electron microscopy and fluorescence microscopy showed that after administration of PLA-DH microspheres, they were rapidly internalized by the structure of the vascular endothelium, as shown in Figure 3, Figure 4, Figure 5 and Figure 6.

### 2.5. Histopathology of the Liver, Heart, Lung and Kidney of Rats after SVPLA Microsphere Administration

Histopathological examination of the liver, heart, lungs and kidneys of the test animals revealed no pathological changes in the internal organs after intra-arterial and intravenous administration of a dispersion of SVPLA microspheres (A, B, C concentrations: group preparations stained with H + E, according to Table 5).

### 2.6. Effect of Microsphere SVPLA Inflammation of the Vessel Wall in an Animal Model (Rat) 24 h, 7 Days and 4 Weeks after Administration

**Figure 7:**
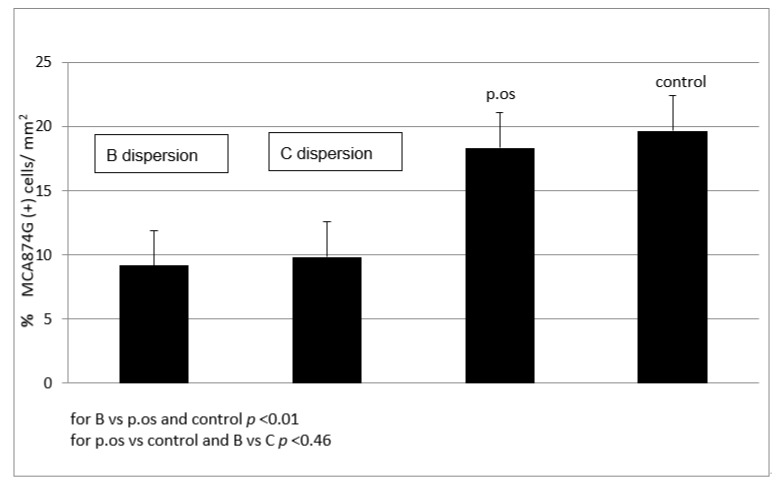
The presence of macrophages in the wall of the aorta after 24 h (groups receiving SVPLA (B and C SVPLA) vs. SV p.os and control). SV dispersion concentration; B: 0.1750 mg/mL, C: 0.0875 mg/mL. SV orally 0.6 mg/kg; Control; saline 0.9% NaCl. Each group *n* = 6; six vascular samples (aorta) were tested per animal (total 36 per group).

Quantitative analysis of macrophages (MP) in the vascular wall of animals, which corresponds to the inflammatory response, 24 h after administration of the 25% SVPLA dispersion showed significantly lower (*p* < 0.01) amounts compared to the control that received PTX perivascular and 5FU and the group receiving SV per os (Figure 7).

In the group receiving 50% SVPLA dispersion, the number of macrophages in the vascular wall was significantly lower (*p* < 0.01) than that in the control group. Comparing the group receiving the SV per os and the control group, there were no significant differences (*p* < 0.46) in the number of macrophages in the wall of the vessel 24 h after the start of observation (Figure 7). There was extensive inflammatory infiltration of eosinophils, lymphocytes and macrophages in adipose tissue surrounding the iliac artery vs. the control group after 24 h of observation.

Quantitative analysis of macrophages in the vascular wall of the animal 7 days after administration of C dispersion of SVPLA showed significantly lower (*p* < 0.01) amounts compared to the control and the group receiving the SV per os. Focal accumulation of foamy macrophages in the intima of the vena cava of the rats was observed in this group.

**Figure 8:**
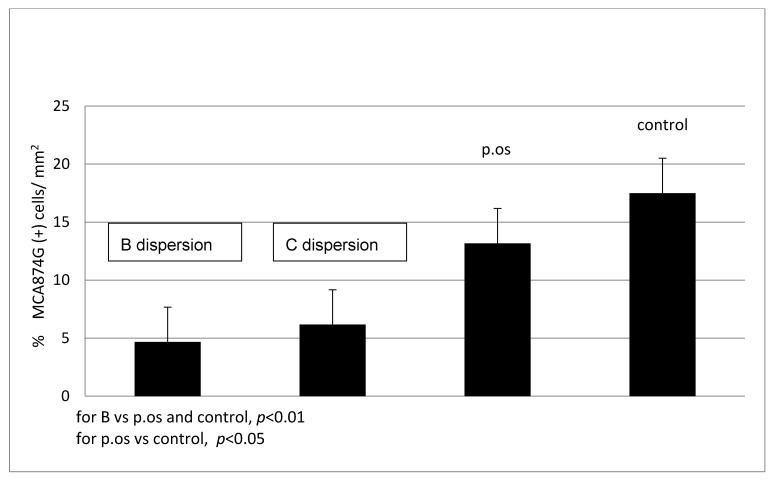
The presence of macrophages in the vessel wall (aorta) after 7 days (groups receiving B and C SVPLA vs. p.os and control). SVPLA dispersion concentration; B: 0.1750 mg/mL, C: 0.0875 mg/mL. SV orally 0.6 mg/kg; Control; saline 0.9% NaCl. Each group *n* = 6; six vascular samples (aorta) were tested per animal (total 36 per group).

In the group receiving the B dispersion of SVPLA, the number of macrophages in the vascular wall was significantly lower (*p* < 0.01) than that in the control group. Comparing a group receiving the oral SV and the control group, no significant differences (*p* < 0.15) in the number of macrophages in the vascular wall 7 days after the start of observation (Figure 8) were observed.

There was extensive inflammatory infiltration of eosinophils, lymphocytes and macrophages in adipose tissue surrounding the iliac artery in the control group after 7 days of observation.

**Figure 9:**
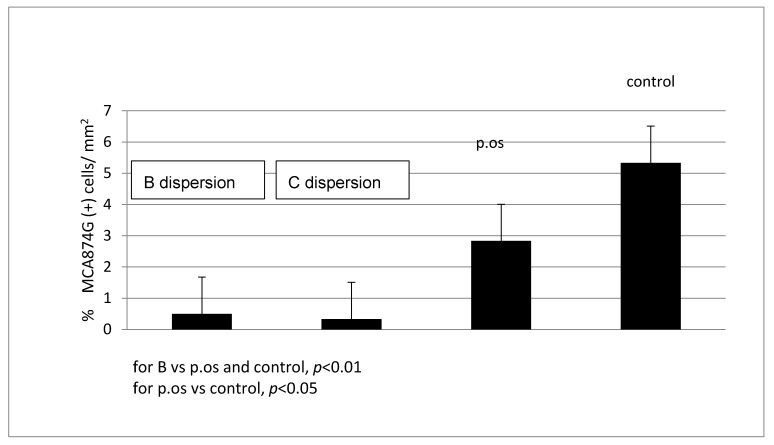
The presence of macrophages in the vessel wall (aorta) after 28 days (groups receiving B and C SVPLA vs. p.os and control). SV dispersion concentration; B: 0.1750 mg/mL, C: 0.0875 mg/mL. SV orally 0.6 mg/kg; Control; saline 0.9% NaCl. Each group *n* = 6; six vascular samples (aorta) were tested per animal (total 36 per group).

After 4 weeks, quantitative analysis of macrophages in the vascular wall in the group of animals that received C SVPLA dispersion showed significantly lower (*p* < 0.01) amounts compared to the control and the group receiving the SV per os (Figure 9). Preparations of the aorta in groups SVPLA C and B showed normal aspects of the vessel wall.

In the group receiving B SVPLA dispersion, the number of macrophages in the vascular wall was significantly lower (*p* < 0.01) than that in the control group. Comparing the group with the oral SV and the control group showed no significant differences (*p* < 0.15) in the number of macrophages in the vessel wall 4 weeks after the start of observation (Figure 9).

Comparing the groups receiving SVPLA (C and B) 24 h, 7 days and 4 weeks after administration of SVPLA microspheres, there were no statistically significant differences in the presence of macrophages in the rat aortic wall.

Total cholesterol levels in the blood serum after 24 h, 7 days and 28 days are shown in Table 3.

## 3. Materials and Methods

### 3.1. Drugs and Other Substances Used in the Studies

Simvastatin (SV) (Polfa Grodzisk, Grodzisk Mazowiecki, Poland), an inhibitor of 3-hydroxy-3-methylglutaryl-coenzyme A (HMG-CoA) with a purity >99%, was used as the loading drug in preparing poly(L,L-lactide) particles (SVPLA). Animals receiving SV in food received it as an additive at a dose of 0.6 mg/kg. Paclitaxel (PTX) (Sindaxel, Sindan, Bucharest, Romania) was in the form of a concentrate for infusion at a concentration of 6 mg/mL. PTX solution was prepared by adding 10 mL of PTX to 90 mL of 0.9% NaCl, and the obtained solution with a concentration of 0.6 mg PTX/mL was used for the experiments. 5-Fluorouracil (5FU) (1000 medac Fluorouracil, Medac, Germany) was used as a solution for intravascular infusion of 50 mg/mL. 5FU solution was prepared by adding 10 mL of 5-FU up to 90 mL 0.9% NaCl. Compounds for particle preparation were as follows: dansylhydrazine (Fluka), L,L-lactide (Purac Biomaterials), poly(vinyl alcohol) with M_w_ = 72,000 (POCh, Gliwice), dichloromethane (Sigma-Aldrich), initiator n-octanol (Sigma-Aldrich), catalyst tin (II) 2-ethylhexanoate (Sigma-Aldrich), and deionized water (ADRONA water purification system).

### 3.2. Poly(L,L-Lactide) Particles Loaded with Simvastatin (SVPLA) and/or Poly(L,L-Lactide) Particles with Fluorescent Dansylhydrazine Used in the Studies

The synthesis of poly(L,L-lactide) from L,L-lactide, characterization of the obtained polymer, preparation of microparticles from poly(L,L-lactide) loaded with simvastatin (SVPLA), determination of simvastatin content and the procedure for preparation of poly(L,L-lactide) particles labeled with fluorescent dansylhydrazine are described in the Supplementary Material.

### 3.3. Determination of the Release of Simvastatin (SV) from SVPLA Particles Investigated In Vitro

The release profile of simvastatin from SVPLA particles using a UV-VIS spectrophotometer (Specord S600, Analytik Jena, Germany) was determined. SVPLA particles (0.50 g) containing 8.1% *w*/*w* simvastatin were suspended in 25 mL 0.15 M PBS (pH 7.4, I = 0.15 M). The sample was incubated at 37 °C. After 1, 25, 51, 171 and 266 h, samples were removed and centrifuged to separate particles from the supernatant. The concentration of simvastatin was determined in supernatants from the registered UV-VIS spectra based on a calibration curve (at λ = 240 nm).

### 3.4. Studies of SVPLA Microsphere Delivery to Biological Material (In Vitro, In Vivo)

The studies using biological material were conducted with the consent of the Bioethics Committee of the Medical University of Lublin.

For injections into animal vessel walls, suspensions of SVPLA particles with variable particle contents at various intervals were used.

Experimental studies were performed in two steps.

Step 1: The pilot study.

Step 2: The effect of simvastatin administered in the form of microspheres (SVPLA) into inflamed vascular endothelium (the main study).

Impact assessment of PLA microparticles labeled with a fluorescent label (PLA-DH) on the microcirculation. Microcirculation model in vivo (the pilot study).

Studies evaluating the effect of microstructures of polylactide (PLA) on microcirculation were performed in animal models (BALB/c mice).

To observe blood flow in vivo, a microparticle cremasteric muscle microcirculation was used.

A microcirculatory study using an in vivo animal model (BALB/c mice) was performed according to the method described by Vicaut and Laemmel [9,10] in the laboratory Hospital Fernand-Vidal, University of Paris VII in Paris. The average weight of mice (*n* = 6) used in the experiments was 28 ± 1.2 g. The mice were fed a standard diet (R/MH, Ssnif, Germany). Animals were anesthetized intraperitoneally with pentobarbital and ventilated through a tracheotomy tube.

The skin was disinfected, and then the right elevator muscle nucleus was exposed through a long, longitudinal incision of the scrotum. Then, after separation from the surrounding tissue using a small metal ring coated with rubber, the muscle was gently spread out so that the ring was in the center of the main artery elevator muscle. Next, the muscle and testicle were gently placed in a special chamber to illuminate the tissue using a source of light condensation. The muscle was perfused with modified Krebs-Henseleit solution containing (mM/L): 118 NaCl, 5.9 KCl, 1.25 CaCl_2_ × 2 H_2_O, 0.5 MgSO_4_ × 7H_2_O, 28 NaHCO_3_ and 10 g of glucose. The temperature of the solution was adjusted to 34.5 °C (muscle cell). The solution was treated with a mixture of gases: 6% CO_2_ and 94% N_2_. The pH of the solution was 7.43 ± 0.03, the partial pressure of oxygen was PO_2_ = 25 ± 1.7 mmHg, and carbon dioxide was PCO_2_ = 40 ± 1.0 mmHg. To optimize the image, a special condenser image was used (Leitz 0.35 S30).

The sample was covered with a Plexiglas plate for isolation from the environment and a backlight lamp 100 W (tungsten-halo) was used. The image was magnified 20× (lens) and 10× (ocular). The total magnification on the screen was 1400×.

Next, the femoral artery was administered 100 microliters of saline via a microcatheter bolus, and microcirculatory flow was observed for 15 min. Then, 100 microliters of a 1:20 solution of the polylactide microspheres containing simvastatin (SVPLA) and a fluorescent dye 1% dansylhydrazine was administered for 30 min at intervals of 5 min and in the microcirculatory flow in the cremaster muscle was observed under a special light microscope (Leitz, Oberkochen, Germany) coupled to a computer (Apple Inc., Cupertino, CA, USA). The flow was evaluated using a digital camera (Panasonic, Osaka, Japan) coupled to a microscope and a Sony S-VHS recorder (Sony, Minato, Japan) with a write speed of 25 frames per second.

To assess blood flow in the microcirculatory, the flow rate of erythrocytes was measured.

The capillary diameter was evaluated by measuring 16–21 capillaries in each animal. Their diameters were calculated, and the results are reported as the average of measurements ± standard deviation (SD).

Additionally, the number of red blood cells flowing through the vessel segment was evaluated per second. The video was recorded at normal and slow speeds. As the registration of movement was not synchronized with the cardiac cycle, the flow rates were averaged based on 50 heart cycles. The flow rate was measured for each animal 50x. Theoretical microvessel hematocrit (Ht) was calculated using Formula (2).

Ht = F_m_ × MCV/V_m_ × π × (D/2)^2^ (2)

where

F_m_—the average velocity of flow of erythrocytes;

MCV—average volume of erythrocytes (49 fl);

V_m_—flow rate of erythrocytes after microsphere administration (mm/s);

D—average diameter of microvessels.

**Figure 10:**
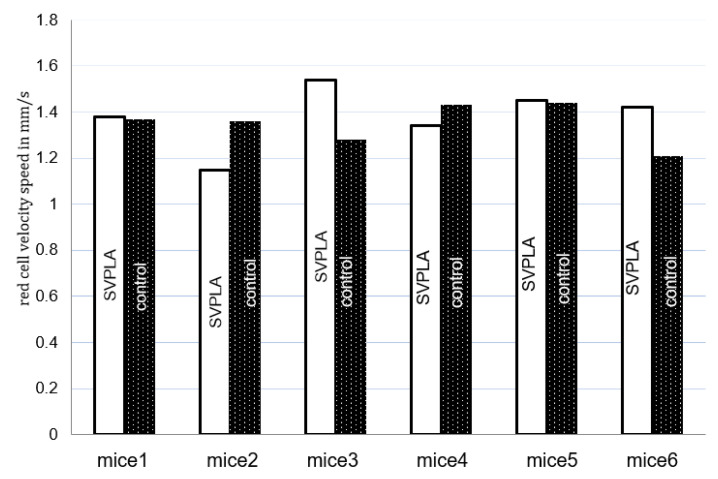
The velocity distribution of erythrocytes after administration of SVPLA microspheres vs. control. Animal model of microcirculation (mice).

In the second stage of the study, muscle microcirculation was illuminated with a mercury lamp and an appropriate set of filters at 20× magnification (960× magnification on the screen). This magnification allowed for the observation of the microcirculation with PLA-DH microspheres using a fluorescence microscope (Leica Epifluorescence, Heidelberg, Germany) (Figure 10).

#### The Effect of Simvastatin Administered in the Form of Microspheres (SVPLA) into Inflamed Vascular Endothelium (the Main Study)

The main study flow chart is presented in Table 4.

We used a modified animal model (rat) for the assessment of inflammation induced by cytostatic factors (PTX/5-FU) [8]. In a pilot microvascular study, we used an animal model (mouse) as described by Laemmel and Vicaut [9,10] and we did not observe any harmful effects on microcirculation in vivo or in vitro [11].

The characteristics of SVPLA suspensions of particles used in the biological studies are presented in Table 5.

Microsphere (SVPLA) dispersion was washed extensively with additionally filtered di. water through a 0.2 μm nylon filter (Nylon Net Filter; Merck Millipore Corp., Burlington, MA, USA) before dispersion was administered to animals.

Under general anesthesia (pentobarbital; 30 mg per kg intraperitoneally), previously disinfected and shaved skin of an animal in the groin area was incised, and the distal part of the aorta was exposed. After 30 s of aorta ligation, the appropriate dispersion of SVPLA was injected into the vessel. After 30 min, the animal was euthanized, and relevant parts of the vessel (aorta or vein) were collected. Vessel samples were suspended in 0.9% NaCl and frozen.

Biological samples were homogenized and then dissolved in 100 mL of acetonitrile and 0.025 M phosphate buffer, pH = 4 (65:35, *v*/*v*) under stirring with a mechanical stirrer for 3 h. A sample volume of 5 mL was centrifuged. The SV vessel wall concentration was analyzed using HPLC (Shimadzu; Japan). The results are presented as the mean SV deposition in the vessel wall ± SD (standard deviation) as a % of the total dose administered to the vessel SV.

### 3.5. Characteristics of Animal Groups

We used 72 rats (Wistar) of both sexes with an average weight of 286 ± 21 g. The rats were randomly assigned to experimental groups with 6 per group.

Animal clinical evaluation was performed two times per day. Depending on the experimental group, animals were anesthetized by intraperitoneal administration of pentobarbital (Morbital^TM^; Biowet, Pulawy, Poland).

All animals were fasted on the day preceding the day of tests. The control groups received SV orally; SV was administered with food 1 time per day at a dose of 0.6 mg/kg (equivalent to 40 mg per day for an adult human weighing 70 kg) for 7 days before the beginning of the study and continued until completion (at 24-h intervals). Abdominal aorta and iliac vein inflammation were induced by administering perivascular vessels 1 mL of PTX (equivalent to 0.6 mg) and 1 mL of 5-FU (corresponding to a dose of 5 mg) for 3 consecutive days preceding the tests.

### 3.6. Deposition Test of PLA Particles Labeled with Fluorescent Dansylhydrazine (PLA-DH) in the Wall of the Vessel

The microspheres were also prepared using a mixture of poly(L,L-lactide) and dansylhydrazine (PLA-DH) (5% by weight to polymer). The procedure for the preparation of dansylhydrazine-containing particles was similar to the procedure leading to nonfluorescent particles, and both recipes are described in Section S2. For this test, a group of 6 rats was used. The PLA-DH particles were administered to the aorta. After collecting vascular samples to avoid deformation of the vascular tissue, collected fragments were positioned retaining their original shape. The material obtained from animals was placed in 10% formalin buffer. Vessel wall samples (lengthening their long axis) were embedded in paraffin blocks. Evaluation of samples was performed by fluorescence microscopy (Nikon Eclipse TE 2000-S, Nikon, Tokyo, Japan).

Sample assessment was performed using scanning electron microscopy (SEM-JSM5500C, LV, JEOL, Tokyo, Japan).

### 3.7. Measurements of Biochemical Parameters in Serum

Blood needed to determine biochemical parameters was collected at autopsy (after 24 h, 7 days and 4 weeks after SVPLA) into sterile tubes to a standard clot. Approximately 1 h after sampling, the blood was centrifuged for 5 min at 4000 rpm at 4 °C in an Eppendorf centrifuge 5804 R (Germany). The supernatant was then transferred to Eppendorf tubes. Until assayed, serum samples were stored in an ultrafreezer (Polar 530V, Angelatoni Industrie, Massa Martana, Italy).

Total cholesterol and triglycerides were determined in the serum. All assays were performed by spectrophotometric methods using a Power Wave XS camera (BioTek Instruments Inc., Winooski, VT, USA). The reagent kits used were from Cormay (Lublin, Poland).

### 3.8. Histopathological and Immunohistochemical Studies

Histopathological and immunohistochemical tissue sections were obtained from the vessel wall material and were analyzed using laboratory facilities and consulting specialists in the Department of Pathology, Medical University of Lublin. During the autopsy, the abdominal aorta with common iliac arteries, lung, kidney, liver and heart were collected. To avoid distortion, the artery material was spread on a corkboard. The material was fixed in 10% buffered formalin for approximately 24 h. The arteries were cut perpendicular to the long axis and, like other segments, embedded in paraffin. Then, the sections were cut and stained with hematoxylin and eosin, and van Gieson and resorcinol-fuchsin staining was performed [12]. The sections were evaluated under an Olympus BX45 light microscope (Japan).

Immunohistochemical staining was performed on paraffin sections of arteries with a thickness of 3 μm. For antigen unmasking, sections were placed in citrate buffer (0.01 M, pH 6.0) and heated in a microwave oven (Samsung; power 750 W) three times for 5 min. Endogenous peroxidase was blocked with 3% hydrogen peroxide solution at room temperature for 5 min. The sections were then incubated with a mouse monoclonal antibody directed against human macrophages, MCA874G (clone MAC387, isotopes IgG1; Serotec), diluted 1:100 for 60 min at room temperature and the DakoEnvision^+ TM^/HRP kit (Dako, Glostrup, Denmark) was used for 30 min at room temperature [12].

After each step, the sections were washed in PBS solution (phosphate buffered saline, pH 7.4). The reaction was visualized with a DAB solution (Dako, Glostrup, Denmark). Then, cell nuclei were counterstained using Mayer hematoxylin until the color reaction was captured. Additionally, control reactions were performed. As suggested by the manufacturer, for the positive control, human spleen sections were used, and for the negative control, antibody was used instead of the corresponding nonimmunized control immunoglobulin. Then, the cells were counted for positive immunohistochemical staining with an antibody test (macrophages) at 20× magnification using a microscopic image analysis system, and the result is presented as the number of cells/mm^2^. For the evaluation of computer-assisted systems, microscopic image analysis was performed using an Axiostar Plus light microscope (Zeiss, Jena, Germany), an Exwave HAD camera (Sony, Minato, Japan) and a PC with Multiscan v 5.1 (CCS, Warsaw, Poland).

### 3.9. Statistical Analysis

The results obtained were subjected to statistical analysis performed using Statistica 10.0 GB (Statsoft Inc., Tulsa, OK, USA).

The range of values (min, max), mean (M) and standard deviation (SD) were calculated. For the characteristics with normal distribution, the significant differences between groups were assessed using one-way ANOVA. For small groups or features that did not have a normal distribution, nonparametric ANOVA, *t*-test and the Mann–Whitney U test were used. Differences with *p* < 0.05 were considered statistically significant.

## 4. Discussion

The present study demonstrated that a PLA loaded with statin DDS might be a drug carrier in local vessel wall treatment [13,14]. The study also confirmed that the SVPLA microspheres undergo internalization in the vessel wall and allow release of the contained drug directly after local administration. Decuzzi et al. noted that the bloodstream in the vessel comprises a paraendothelial blood current that is partly free from large cellular components [15]. SVPLA microspheres, with random movement across the bloodstream, have a better chance of contacting the vascular endothelial surface [15,16]. In our study, after ia/iv administration of SVPLA microspheres, a high level of free SV in the vessel wall was achieved (Figure 3; Table 2). The level of SV in the vessel wall exceeded the maximum level reached in the blood plasma observed when SV was administered at doses equal to 80 mg/day orally in humans, which ranges from 10 to 34 ng/mL plasma [17]. Generally, the target tissue for statins class is the liver, not vessel wall. The final statins effect is lowering LDL in the hepatic circulation. The pleiotropic effect of statins is anti-inflammatory effect observed at vessels but at very high oral doses (and after several months of treatment). Unfortunately, these high oral doses are not well tolerated by many patients (7–29%) and make side effects possible (from muscle pain up to rhabdomyolysis). For these reasons we tested our hypothesis [18]. This result indicates that using SVPLA microspheres as local DDSs (distributed in the range of several cm of the vessel length) is possible and leads to high local concentrations of SV (like other statins). This finding presents a treatment opportunity after percutaneous angioplasty of selected vessel (arterial) segments (as procedural, single-dose DDS at the end of PTA). Thus, the effect of plasma statins after oral administration is limited to a relatively short time because statins are subjected to rapid uptake by the liver and enter the hepatic circulation after the first hepatic pass [19]. This DDS is not an oral statin competitor but rather a different approach for specific clinical situations, such as peripheral PTA.

During the disintegration of SVPLA, free SV (as a form of the inactive lactone to the active form of the β-hydroxy acid) was released. Due to the slower activation of the SV in the vessel wall compared to liver cells and its further local metabolism, a single administration of SVPLA microspheres to the vessel wall can provide long-term local effects of the drug.

A high concentration of SV after a single administration supports the hypothesis that a relatively sufficient portion of the SVPLA microspheres undergo phagocytosis and internalization in vascular endothelial cells. Due to its average size (approximately 6 µm), excess SVPLA undergoes rapid uptake by reticular endothelial liver cells (RES) according to previous research [20]. SVPLA microspheres that were not absorbed on the vessel wall (after local administration) were rapidly cleared from the blood circulation in the liver [21,22]. Local drug delivery using SVPLA microspheres is advantageous because it allows slower, local drug release and action without general side effects.

Mechanical damage during PTA (shear, pressure surges) and inflammatory conditions may cause disadvantageous effects, such as the destabilization of the plaque, micro bleeding inside the vessel and the entire cascade leading to the formation of intravascular thrombus formation. A thrombus can cause acute coronary syndrome or peripheral vessel-dependent ischemia (PTA), and it is potentially a dangerous clinical situation [3].

Our results showed that iatrogenic vessel wall inflammation in experimental animals (which could be compared with mechanical injury after PTA) underwent rapid healing in the group receiving SVPLA microspheres compared to the control group, irrespective of observation time (24 h, 7 days and 4 weeks), as evidenced by a significant reduction in the numbers of macrophages observed in the vessel wall. Additionally, a group of animals receiving only food containing SV also showed a reduction in the inflammatory reaction in the aortic wall relative to controls, but it was less marked and visible after 7 days of observation (*p* < 0.05). Nevertheless, each group receiving SVPLA microspheres showed a significant reduction in the inflammatory reaction compared to the group receiving SV food (*p* < 0.01). In contrast, in the control group, an increased (compared to the group receiving the SVPLA microspheres) number of macrophages in the vessel wall was maintained throughout the observation period. Despite a broad particle size distribution (D_w_/D_n_ = 1.68), the therapeutic effect of SV loaded in PLA particles locally injected after a single dose was achieved.

Administration of the drug was limited to SVPLA particles locally, and the effect was independent of the effects of cholesterol in blood serum. In biochemical assays, administration of SVPLA did not meaningfully alter the levels of cholesterol in the blood serum during the entire study period. In the group receiving SV per os, a significant reduction of approximately 20% in serum cholesterol levels was observed. Both in the control group and in the group receiving SVPLA, there was no significant change in the levels of cholesterol or triglycerides. Therefore, the anti-inflammatory effects of SV given in the form of microspheres directly into the vessel wall cannot be explained by its action on the lipid profile. The greatest reduction in the inflammatory response in the vessel wall was observed in the groups receiving SVPLA microspheres (with no significant changes in serum cholesterol) rather than in the group receiving SV food (in which a significant reduction in serum cholesterol in the blood was observed). The most likely explanation is that the pleiotropic effects of simvastatin were released from the microspheres directly in the vessel wall, independent of changes in the lipid profile. Compared to SV received with food, a reduction in inflammation (expressed as a reduction in the number of macrophages) occurred only after 24 h of administration of SVPLA. However, in the group receiving SV with food, a reduction in the inflammatory response was observed 7 days and 4 weeks after the start of the study compared to controls. Compared to SVPLA, SV given with food was significantly weaker (*p* < 0.01), despite a considerable reduction in cholesterol levels in blood serum. The studies demonstrated that once administered to the vessel, simvastatin achieved a high concentration in the vessel wall, a slow and gradual release caused a long-lasting local therapeutic effect, independent of its action on the lipid profile.

Based on the results of our study, we hypothesize that one statin DDS injection, such as after PTA facing long vascular stenosis, long balloon inflations and finally long vascular injury (calculated for several cm), could improve the PTA result. Microspheres loaded with SV have potential due to their local action and minimal systemic effects. Long, diffuse, peripheral atherosclerotic lesions with inflammatory components may be a target for statin DDS therapy with microspheres, especially where long drug-eluting stents are difficult to implant (such as the popliteal artery) [22]. Lesions in peripheral artery disease appear to be the first choice for this DDS. The procedure that has been elaborated will be developed in future studies with PLLA DDSs with statins for local vascular therapy.

## 5. Conclusions

Possible formulation of the therapeutic procedure concept, the DDS with statins with specific indications in cardiology (single dose after PTA), seems to be an interesting approach to test in humans. This DDS is not an oral statin competitor but rather a different approach for specific clinical situations, such as peripheral PTA. Local (peripheral artery for this microsphere diameter), targeted DDS with minimal general side effects has great potential for patients with diffuse atherosclerosis and complex localization, such as the popliteal artery.

## Figures and Tables

**Table 1:** SVPLA particles chart.

Parameter	The Microspheres Used in the Study
The number of microspheres measured, N	301
The largest microsphere measured, μm	17.0
The smallest microsphere measured, μm	0.22
The average diameter of the microspheres, μm (± SD)	6.40 ± 3.28

**Table 2:** Concentration of SV in the vessel wall after the administration of the SVPLA dispersion into the rat aorta.

Sample Number	SVPLA A (μg/mg) ^1^	SVPLA B (μg/mg)	SVPLA C (μg/mg)	Control ^2^ SV per os
1	18.34	27.21	10.47	0
2	8.58	16.74	8.58	0
3	33.15	27.21	11.15	0
4	23.60	30.72	16.22	0
5	23.11	28.25	15.01	0
6	24.96	39.04	24.41	0
Average ± SD	21.95 ± 8.12	28.19 ± 7.17	14.30 ± 5.72	0

^1^—SV concentration in micrograms per mg weight of the vessel wall. ^2^—SV per os, 0.571 mg/kg-non detectable.

**Table 3:** Total cholesterol levels (mmol/L) in rat blood serum, depending on the time scale of the sampling: * *p* < 0.01 vs. control; ** *p* < 0.01 versus control: # all groups *n* = 6.

Group #	T	Min	Max	Mean ± SD
SVPLA 25%	24 h	1.12	1.76	1.26 ± 0.21 *
	7 days	1.08	1.54	1.24 ± 0.16 *
	28 days	0.99	1.32	1.21 ± 0.21 *
SVPLA 50%	24 h	1.01	1.97	1.29 ± 0.28 *
	7 days	0.99	1.87	1.31 ± 0.31 *
	28 days	0.96	1.45	1.18 ± 0.21 *
SV with food	24 h	0.91	1.43	1.09 ± 0.18
	7 days	0.80	1.21	0.94 ± 0.14 **
	28 days	0.76	0.98	0.86 ± 0.14 **
Control	24 h	1.09	1.68	1.22 ± 0.12
	7 days	1.08	1.78	1.30 ± 0.21
	28 days	1.02	1.57	1.23 ± 0.21

**Table 4:** Main study flow chart.

Main Study Flow Chart				
Day-3, 2, 1	Para Vessel PTX/5-FU Injections *n* = 72			
Day 0	SVPLA 50% *n* = 18	SVPLA 25% *n* = 18	SV * (0.58 mg/kg) *n* = 18	Control *n* = 18
24 h ***	MP ** (vessel wall) *n* = 6	MP (vessel wall) *n* = 6	MP (vessel wall) *n* = 6	MP (vessel wall) *n* = 6
7 Days	MP (vessel wall) *n* = 6	MP (vessel wall) *n* = 6	MP (vessel wall) *n* = 6	MP (vessel wall) *n* = 6
4 Weeks	MP (vessel wall) *n* = 6	MP (vessel wall) *n* = 6	MP (vessel wall) *n* = 6	MP (vessel wall) *n* = 6

N—number of tested animals; * with food; ** MP—macrophage count and assessment; *** blood assessment for biochemical parameters.

**Table 5:** SVPLA particles dispersion characteristics used in biological studies.

	SVPLA Dispersion Concentration (mg/mL)	Recipe	SV Loading Dose/kg	SV Solution Volume Administered (mL) per Animal
Solution A	0.3500	1 mL A	0.58 mg	1
Solution B	0.1750	1 mL A + 1 mL PBS	0.58 mg	2
Solution C	0.0875	1 mL A + 3 mL PBS	0.58 mg	3

## Supplementary Materials

The following are available online at https://www.mdpi.com/article/10.3390/ijms22147486/s1. Supplementary materials describe the synthesis of poly(L,L-lactide) from L,L-lactide, the characterization of the obtained polymer, the preparation of particles loaded with simvastatin (SVPLA), the determination of simvastatin content (Section S1) and the procedure for poly(L,L-lactide) particles labeled with fluorescent dansylhydrazine (PLA-DH) (Section S2).
