# Supplementary material for: Anti-Inflammatory Effect of Very High Dose Local Vessel Wall Statin Administration: Poly(L,L-Lactide) Biodegradable Microspheres with Simvastatin for Drug Delivery System (DDS)
Source: Int J Mol Sci. 2021 Jul 13;22(14):7486. doi: 10.3390/ijms22147486 (PMC8303821; doi:10.3390/ijms22147486)
Supplement: Supplementary file 1 [file ijms-22-07486-s001.zip › ijms-1252665-supplementary.pdf]

Supplementary material.

Anti-inflammatory Effect of Very High Dose Local Vessel Wall Statin Administration: Poly(L,L-lactide) Biodegradable Microspheres with Simvastatin for Drug Delivery System (DDS)

Piotr Wacinski, Mariusz Gadzinowski, Wojciech Dabrowski, Justyna Szumillo, Jakub Wacinski, Nathalie Oru, Eric Vicaut, Stanislaw Czuczwar, Janusz Kocki, Teresa Basinska, Andrzej Wysokiński and Stanislaw Slomkowski

1. Poly(L,L-lactide) microspheres loaded with simvastatin used in the studies

This section comprises description of synthesis poly(L,L-lactide) from L,L-lactide, characterization of obtained polymer, preparation of particles loaded with simvastatin, determination of simvastatin content.

1.1 Preparation of poly(L,L-lactide) microspheres loaded with simvastatin (SVPLA).

1.1.1 Synthesis of poly(L,L-lactide).

Poly(L,L-lactide) (PLA) with target molecular weight M_n = 10 000 g/mol was synthesized by bulk ring-opening polymerization. 10,0 g (0.069 mol) of L,L-lactide (Purac Biomaterials, Holland), 0.065 g ($5x10^{-4}$ mol) of n-octanol (Sigma-Aldrich) as an initiator and tin(II) 2-ethylhexanoate (Sigma-Aldrich) as a catalyst (10 mol % in relation to the initiator) was sealed in an ampoule under vacuum and carried out at 130°C for 8 hours. After this time, the reaction vial was cooled to the room temperature and opened. Number average molecular weight of poly(L,L-lactide) was equal 10600 g/mol (M_w/M_n=1.38) and was determined by GPC analysis at the following conditions: flow of dichloromethane 0.8 mL/min, columns: PLGel 5 microns, detectors: MALLS - Wyatt Heleos II and DRI – Wyatt Optilab T-Rex.

1.1.2 Microparticles preparation from poly(L,L-lactide).

Particles loaded with simvastatin were obtained by classical solvent evaporation method. Poly (L,L-lactide), and simvastatin (Polfa Grodzisk, Poland) 10% by weight relative to the polymer were dissolved in dichloromethane and added to 1% aqueous solution of polyvinyl alcohol (POCh Gliwice, Poland) with vigorous stirring using homogenizer (MPW 120, Mechanika Precyzyjna, Warszawa) for 2 min. at room temp. with maximum speed. Thus, the obtained aqueous emulsion of poly (L,L-lactide) and simvastatin in dichloromethane was stirred with a low speed of stirrer for 48 hours at 35°C. The resulting particles were centrifuged and washed three times with deionized water (Adrona deionizer). The size of particles was determined by

Scaning Electron Microscopy (SEM) imaging using Jeol 5500LV microscope. Representative microphotograph of microparticles is presented in Fig. S1.

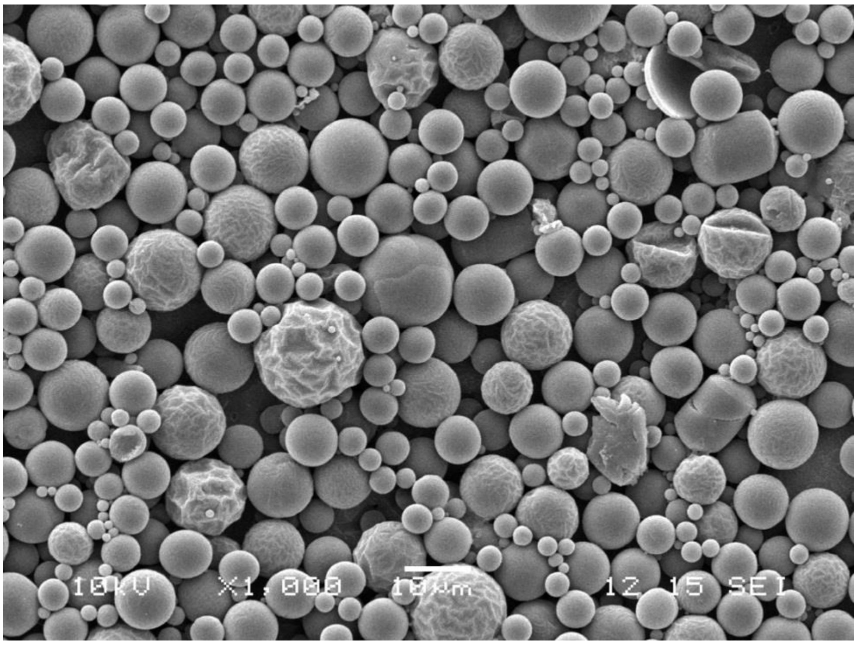

Figure S1. SEM image of poly(L,L-lactide) microspheres loaded with simvastatin.

Size and size distribution of microspheres was determined using SigmaScan (Systat) image analysis software. More than 300 particles were sized and statistical analysis was performed as illustrated in Figure S2.

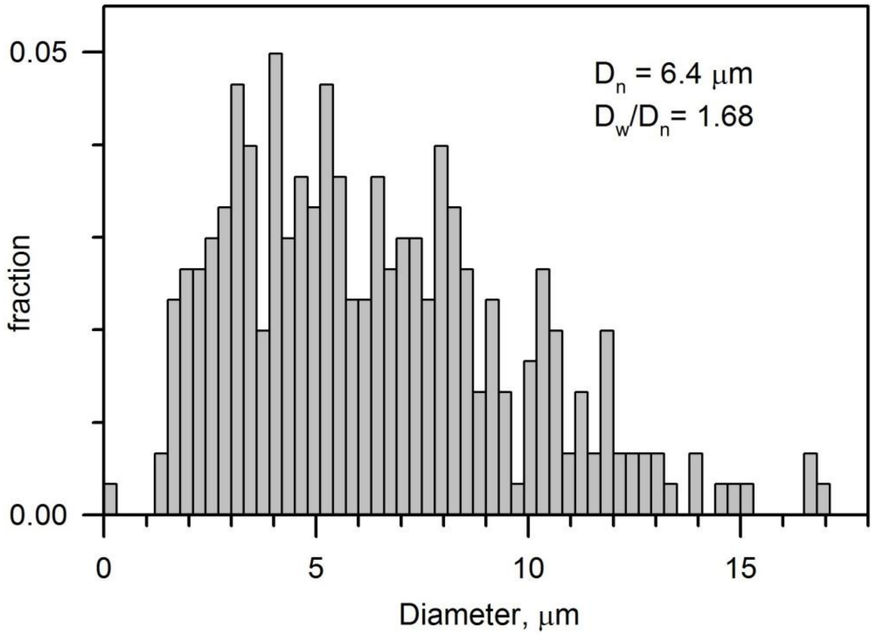

Figure S2. Number average diameter (D_n) and diameter distribution (D_w/D_n) of SVPLA microspheres.

1.1.3 Determination of simvastatin content in SVPLA particles

Simvastatin content in microspheres was determined by ^{1}H-NMR analysis. Microspheres were dried under vacuum, dissolved in chloroform-d and 1H-NMR analysis was performed (Bruker Avance 200 MHz spectrometer). Based on integration signals deriving from simvastatin and polylactide, we found that the ratio of simvastatin in relation to microspheres is equal 7.5 – 8.5 w%. The simvastatin content in microspheres slightly varied from sample to sample.

2. Preparation of poly(L,L-lactide) microspheres loaded with dansylhydrazine (PLA-DH)

This section contains procedure for poly(L,L-lactide) particles labeled with fluorescent dansylhydrazine (PLA-DH).

Dansylhydrazine labeled microspheres were obtained by classical solvent evaporation method. Poly(L,L-lactide), and dansylhydrazine (5% by weight relative to the polymer) were dissolved in dichloromethane and added to 1% aqueous solution of poly(vinyl alcohol) (POCh Gliwice, Poland) with vigorous stirring with a homogenizer. Then, obtained aqueous emulsion of poly-L,L-lactide solution and dansylhydrazine in dichloromethane was stirred with a low speed mixer for 48 hours at 35 °C. The resulting particles were centrifuged and washed three times with distilled water. Particles were observed using optical fluorescence microscope (Nikon Eclipse TE 2000-S, Japan).
